# Dosimetric properties of a beam quality‐matched 6 MV unflattened photon beam

**DOI:** 10.1120/jacmp.v13i4.3701

**Published:** 2012-07-05

**Authors:** Yunfei Huang, R. Alfredo Siochi, John E. Bayouth

**Affiliations:** ^1^ Department of Radiation Oncology Division of Medical Physics University of Iowa Hospitals and Clinics Iowa City Iowa 52242 USA

**Keywords:** flattening filter free, unflattened photon beam, beam characteristics, Siemens Linac

## Abstract

The purpose of this study was to report the characteristics of an equivalent quality unflattened (eqUF) photon beam in clinical implementation and to provide a generalized method to describe unflattened (UF) photon beam profiles. An unflattened photon beam with a beam quality equivalent to the corresponding flat 6 MV photon beam (WF) was obtained by removing the flattening filter from a Siemens ONCOR Avant‐Garde linear accelerator and adjusting the photon energy. A method independent from the WF beam profile was presented to describe UF beam profiles and other selected beam characteristics were examined. The short‐term beam stability was examined by dynamic beam profiles, recorded every 0.072 s in static and gated delivery, and the long‐term stability was evidenced by the five‐year clinical quality assurance records. The dose rate was raised fivefold using the eqUF beam. The depth of maximum dose ($d_{\max}$) shifted 3 mm deeper, but the percent depth dose beyond $d_{\max}$ was very similar to that of the WF beam. The surface dose and out‐of‐field dose were lower, but the penumbra was slightly wider. The variation in head scatter and phantom scatter with changes in field size was smaller; the variation in the profile shape with change in depth was also smaller. The eqUF beam is stable 0.072 s after the beam is turned on, and the five‐year beam stability was comparable to that of the WF beam. A fivefold dose rate increase was observed in the eqUF beam with similar beam characteristics to other reported UF beam data except for a deeper $d_{\max}$ and a slightly wider penumbra. The initial and long‐term stability of the eqUF beam profile is on parity with the WF beam. The UF beam profile can be described in the generalized method independently without relying on the WF beam profile.

PACS number: 87.55.de

## I. INTRODUCTION

Many radiotherapy technologies in use today such as intensity‐modulated radiotherapy (IMRT), stereotactic body radiotherapy (SBRT), and gated treatments prolong treatment time. One way to reduce the treatment time is to increase the dose rate by removing the flattening filter. Historically, a flat photon beam was produced with a flattening filter (WF) to simplify treatment planning in radiotherapy. When treating with an unflattened (UF) beam, the cross‐axis beam profile is conically shaped, a characteristic that can be modeled by computer‐based treatment planning systems. In light of the successful use of UF beams in stereotactic radiosurgery (SRS),^(^ ^1^ ^,^ ^2^ ^)^ there is a greater interest in expanding the use of UF beams to reduce treatment times when using other radiotherapy techniques.^(^ ^3^ ^–^ ^8^ ^)^

A dose‐rate increase of about twofold for 6 MV photon beams was observed in linear accelerators from various vendors^(^ ^9^ ^,^ ^10^ ^)^ by removing the flattening filter. Previous UF beam studies reported changes in beam characteristics such as the energy spectrum and lateral photon fluence fall‐off. Various other advantages, such as a reduction in head scatter and electron contamination, were also reported.^(^ ^7^ ^,^ ^9^ ^,^ ^11^ ^–^ ^21^ ^)^

Removal of the flattening filter increases the weight of low‐energy photon fluence and results in decreased UF beam energy.^(^ ^7^ ^,^ ^16^ ^,^ ^22^ ^)^ The decreased UF beam energy could be increased to the original WF beam energy by matching the percent depth dose (%dd) to that of the WF beam. The dose rate increase and other dosimetric characteristics of an unflattened photon with its energy matched to the original 6 MV WF beam are the focus of this paper. This work describes a method to adjust and calibrate an unflattened photon beam obtained from a conventional 6 MV photon beam line. The UF beam quality specified by the photon component of the percent depth dose at 10 cm, %dd(10)x as defined in TG‐51,^(^ ^23^ ^)^ is tuned to be equivalent of the original 6 MV WF beam. The resulting photon beam is now called the equivalent quality UF beam (eqUF), and selected eqUF beam characteristics were investigated and compared to the conventional WF beam.

Another concern is the lack of a standardized description of the lateral regions in the unflattened cross‐axis profiles.^(^ ^24^ ^)^ The conventional way of identifying the primary beam, field size or penumbra using the 80%, 50%, and 20% points for the flat beam profile does not apply here due to the large dose fall‐off away from the central axis (CAX). An alternative approach presented by Pönisch et al.^(^ ^17^ ^)^ retained the conventional description for UF beam profiles by renormalizing the unflattened beam profile to the inflection point using the WF to UF dose ratio. As clinics increasingly implement UF beams for treatment, a technique for describing the UF beam profile without relying on the WF beam profile may be useful. In this paper, we modify the method presented by Pönisch et al. to describe the unflattened photon beam profile so that it does not rely on the corresponding WF beam profiles, while retaining the conventional use of the 20%, 50%, and 80% points.

Finally, we describe the clinical stability and performance data, paying particular attention to the run‐up characteristics of the beam. Beam performance during the initial 0.2 s of beam on may be more relevant at higher dose rates, since substantial amounts of radiation dose can be delivered prior to feedback from the control system. This paper characterizes the initial (<0.2s) and long‐term stability (over five years) of the UF beam in comparison to the conventional WF beam on the same linear accelerator.

## II. MATERIALS AND METHODS

### A. Beam quality matching

Photon beams were obtained from a dual photon energy standing‐wave accelerator,^(^ ^25^ ^)^ Siemens ONCOR Avant‐Garde linear accelerator (Siemens Medical Solutions, Concord, CA). The first photon energy was a conventional 6 MV photon beam with the flattening filter in place, while the second was a 6 MV photon beam with the flattening filter removed. The beam control parameters utilized by the 6 MV WF beam for this digitally controlled linac were copied over and utilized by the UF beam. These parameters included injection voltage (INJE) and injection current (INJI), bending magnet current (BMI), pulse forming network (PFN), and automatic frequency control (AFC), to name a few. This allowed us to test the impact of simply removing the flattening filter. The %dd curves of a $10\times 10\;{\text{cm}}^{2}$ field in the WF and UF beams under standard conditions were collected with a PTW 0.125 cc Semiflex Type 31010 ionization chamber in a PTW MP3 water tank (PTW Freiburg, Germany) filled with water, and compared. The dose rate of this energy unaltered UF beam was measured and the degree of dose rate increase was determined.

Subsequently, the photon energy of the UF beam was tuned to match the beam quality of the original 6 MV WF beam. Since the study presented by Xiong and Rogers^(^ ^26^ ^)^ illustrates that %dd(10)x is a good indicator of beam quality for flattened as well as unflattened beams, the quality matching process was performed to achieve equivalent values of %dd(10)x.^(^ ^23^ ^)^ Tuning of the photon energy was accomplished by adjusting the beam control parameters of the UF beam. The BMI in the UF beam was increased to select the higher energy electrons so that the UF beam quality matched the WF beam quality. Other beam control parameters, such as the PFN, were finely tuned to optimize the wave guide efficiency at the selected energy level. The INJI was decreased to avoid the saturation of the monitor unit ionization chamber in the treatment head while maximizing output. The dose rate of this eqUF beam was measured and compared to that of the WF beam, as well as to the energy unaltered UF beam.

The degree of equivalence or difference between the WF and eqUF beams was further evaluated by comparison of the overall shape of the %dd curves of the $10\times 10\;{\text{cm}}^{2}$ field using percent difference as well as the gamma index. Additional %dd data were measured with a parallel plane ion chamber (PTW Markus chamber Type 23343) to identify the dose in the buildup region. Measurements were started at a depth of 20 mm moving towards the surface, where a depth of 2 mm was the shallowest depth measured and is referred to as the surface dose in this study. The change in the depth of maximum absorbed dose ($d_{\max}$) associated with the eqUF beam was compared to the original WF beam, as well as to the energy unaltered UF beam.

### B. Beam characteristics

The %dd data and cross‐axis beam profiles for various field sizes ranging from $3\times 3\;{\text{cm}}^{2}$ to $40\times 40\;{\text{cm}}^{2}$ were collected with the same 0.125 cc ion chamber in water. Selected characteristics of the eqUF photon beam were investigated and the change from those of the WF beam quantified. The variation of %dd in various depths, %dd(0.5), %dd(10), and %dd(20), associated with the change in field sizes, were examined and compared between WF and eqUF beams.

The relative in‐air scatter factor — also called the head scatter or collimator scatter factor (Sc) — was measured in air with the same 0.125 cc ion chamber with buildup cap (PTW T31013.3.104 PMMA 6–8 MV) for various field sizes. Total scatter factor was measured at the same distance from the source ($100\;\text{cm}+d_{\max}$) at depth of maximum dose in water for the corresponding field sizes. Phantom scatter factor (Sp) was then obtained by removing the collimator scatter from the total scatter.

The cross‐axis profiles of the eqUF beam were compared to the WF profiles for various field sizes at a depth of 10 cm, using a modification of the method presented by Pönisch et al. to identify lateral regions for UF beam profiles. A dose twice that of the UF beam dose at the inflection point of the dose profile was used as the reference dose, instead of relying on the WF to UF dose ratio to identify the lateral regions for the UF beam profiles. As in the case of the WF beam, (1) the field size can be identified by points with 50% of the reference dose; (2) the penumbra can be identified by points with 20% and 80% of the reference doses; (3) the primary region is identified as the central 80% of the field size; and (4) the out‐of‐field region is outside 120% of the field size. Doses in different lateral regions were then compared between WF and eqUF beams.

The variation in the shape of beam profiles (the combined effects of beam divergence, beam hardening and off‐axis softening) associated with depth was evaluated by the off‐axis ratio (OAR) in a $10\times 10\;{\text{cm}}^{2}$ field. The OAR was also calculated with beam divergence removed to compare the effects of off‐axis softening only over a range of depths.

### C. Beam stability

The stability of the eqUF beam was evaluated both during the initial moments of beam delivery and over an extended period of time. Initial stability (<0.2s) was considered for respiratory‐gated and IMRT applications. The initial stability was evaluated by examining the cross‐axis beam profile during a delivery of 100 MU with the eqUF beam at 1500 MU/min. The cross‐axis profiles of a $25\times 25\;{\text{cm}}^{2}$ field with 0.5 cm spatial resolution were collected with a linear diode array (Profiler, Sun Nuclear, Melbourne, FL), which was able to update the profile data up to every 0.072 s (frame) dynamically. The dose profile recorded in the first two frames was compared to the average profile over the delivery of 100 MU. In addition, the stability of the eqUF beam profile was examined during delivery of 100 MU in gating mode with a 0.5 s gating window to check the ramp‐up effects caused by frequent beam on and off cycles. The profiles in gated delivery were also compared to the average profile under static delivery, and the profile variations evaluated.

Long‐term stability refers to the daily output and dose profile constancy QA records for the eqUF beam since our first clinical use of the beam for stereotactic radiosurgery (SRS) in 2005. The output consistency and beam symmetry of the eqUF beam were compared with the WF beam results during the same setup for the two beams: $25\times 25\;{\text{cm}}^{2}$, 95 cm source‐to‐surface distance, radiation detection system (Tracker, Keithley Instruments Inc., Cleveland, OH) at 5 cm depth in polystyrene, with 5 cm polystyrene below the detector for back‐scatter. The radiation detector contained five ionization chambers: one on the central axis, and four orthogonally placed ionization chambers on a 10 cm radius from the central chamber.

## III. RESULTS

### A. Beam quality matching

**Figure 1:**
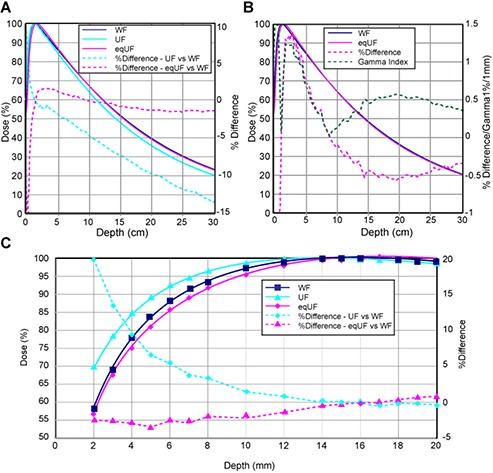
Comparison (a) of %dd measured with 0.125 cc ion chamber in water with a field size of $10\times 10\;{\text{cm}}^{2}$ between WF, UF and eqUF photon beams; (b) between WF and eqUF photon beams; and (c) %dd for depth 2–20 mm measured with Markus plane‐parallel ion chamber.

When the flattening filter was removed without changing the beam control parameters, the %dd curve changed greatly, as shown in Fig. 1(a). The %dd values for the UF beam were 5% and 10% lower than the WF values at depths of 10 cm and 20 cm, thus demonstrating an apparent reduction in beam energy. The beam energy of the unaltered UF beam, %dd(10)x, decreased from 67.1% to 63.8%. The depth of maximum dose, $d_{\max}$, shifted towards the surface by about 2 mm, from 15 mm to 13 mm.

The dose rate in the UF beam obtained by simply removing the flattening filter increased by a factor of 2, compared to the dose rate in the original WF beam.

A 22.5% increase in the BMI and a 25.5% reduction in INJI were made to achieve eqUF beam quality matching as well as to avoid the saturation of the ion chamber monitor while maximizing the dose rate. The percent depth dose at 10 cm depth for a $10\times 10\;{\text{cm}}^{2}$ field was 67% for the eqUF beam, equivalent to the percent depth dose of the WF beam, as shown in Fig. 1(a). The depth of maximum dose, $d_{\max}$, was deeper by about 3 mm when the eqUF beam quality was matched to be within 1% difference from the WF beam quality.

In addition to being matched at 10 cm depth, the %dd curve of the WF beam and eqUF beam were very similar to each other, as seen in Fig. 1(b). The percent difference in %dd between the WF and eqUF beam was all within 1% beyond the depth of about 5 cm. As the depth decreases, the percent difference increases but remains less than 1.5% until reaching the buildup region. In contrast to the result that the %dd in the energy unaltered UF beam was much higher than the original WF beam in the buildup region, the %dd in the eqUF beam was slightly lower, as shown in Fig. 1(c). The surface dose identified at a depth of 2 mm was 20% higher in the energy unaltered UF beam than in the WF beam, but 2.5% lower in the eqUF beam than the WF beam.

The dose rate increase in the eqUF beam was observed to be fivefold: from 300 MU/min in the WF beam to 1500 MU/min in the eqUF beam.

### B. Beam characteristics

**Figure 2:**
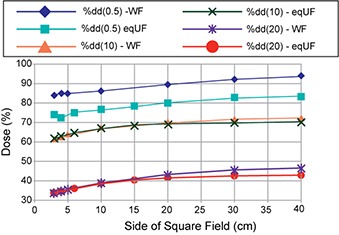
Comparison of %dd at depth of 0.5, 10, and 20 cm between WF and eqUF photon beams

**Figure 3:**
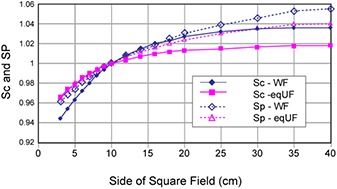
Comparison of collimator scatter factors (Sc) and phantom scatter factor (Sp) between the WF and eqUF photon beams.

The eqUF beams produced substantially less scatter compared to the WF beams. The variations of %dd(0.5), %dd(10), %dd(20), Sc, and Sp, associated with the change in field sizes are shown in Figs. 2 and 3. Overall, less variation with changes in field sizes was observed in all these results for the eqUF beam compared to the WF beam. When the field size is increased from $3\times 3\;{\text{cm}}^{2}$ to $40\times 40\;{\text{cm}}^{2}$, the eqUF beam had 21.7% and 26.2% less variation in %dd at depths of 10 cm and 20 cm, respectively. The variation in %dd at a depth of 0.5 cm was also less in the case of the eqUF beam but to a more modest extent of about 5.2%. The variation of collimator scatter factors associated with the same amount of changes in field sizes was observed to be 9.2% in the WF beam and 5.2% variation for the eqUF beam, indicating 43.3% less variation in the eqUF beam than in the WF beam. Similar variations in phantom scatter factors were also observed: about 9.5% in the WF beam and 7.5% in the eqUF beam, which was about 21.0% less variation in the eqUF beam.

**Figure 4:**
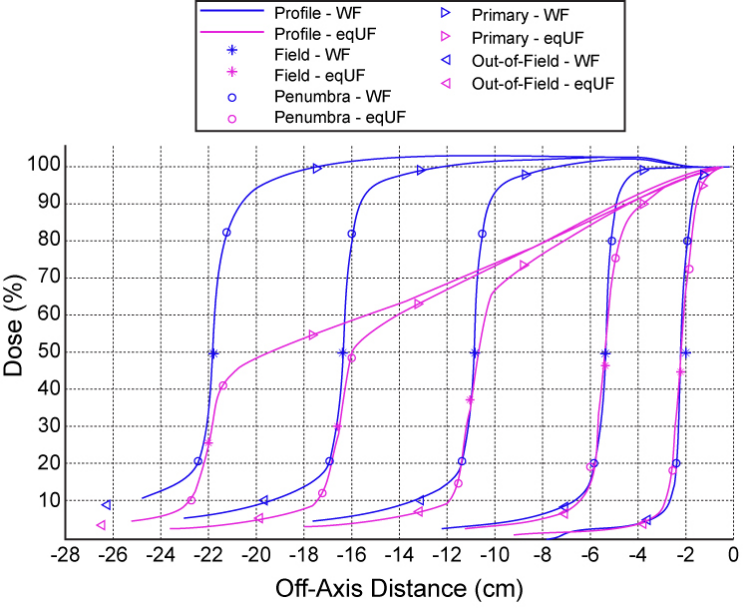
Comparison of cross‐axis profiles of field size 4, 10, 20, 30, and 40 cm of WF and eqUF photon beams at depth of 10 cm.

Figure 4 shows the lateral regions of the eqUF beam profiles identified independently using the reference dose. The field size defined by the width of 50% of the dose at CAX in the WF case and 50% of the reference dose in the eqUF case agreed within 1 mm on average (STD=0.8 mm). The penumbra in the eqUF beams was observed to be 2.4 mm wider on average (STD=0.7 mm) than in the WF beam. Note that the penumbra reported here suffers from the volume averaging over the 5.5 mm cross‐sectional diameter of the 0.125 cc ion chamber, so that it may be slightly larger than in reality for both UF and eqUF beams. The dose in the out‐of‐field region was lower in the eqUF beam, ranging from 13.5% to 61.2% lower for field sizes ranging from 4× 4 to $40\times 40\;{\text{cm}}^{2}$.

**Figure 5:**
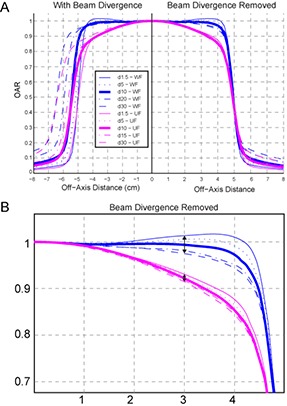
Comparison (a) of beam profiles at various depths with/without the beam divergence removed between the WF and eqUF beams. Variation in OAR (b) associated with the same change in depth is 2.6 times larger in WF beams than in eqUF beams at 3 cm lateral distance.

The off‐axis ratio (OAR) of a $10\times 10\;{\text{cm}}^{2}$ field at different depths calculated with and without the beam divergence removed demonstrates smaller off‐axis softening effect for the eqUF beam. Figure 5(a) shows that the sloped dose profiles from the eqUF beam have less variation in profile shape for the range of measurement depths. The left half of Fig. 5(a) shows profiles without removing the beam divergence. The right half shows profiles with beam divergence removed so that the variation in profile shapes were caused by off‐axis softening effects only. At an off‐axis distance of 3 cm, the variation in OAR in the WF beam was observed to be 2.6 times larger than that in the eqUF beam associated with the same amount of change in depth, as shown in Fig. 5(b).

### C. Beam stability

**Figure 6:**
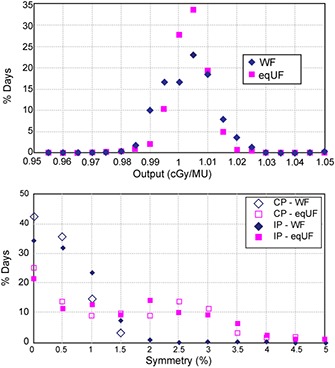
Results of daily QA on output constancy (top) for WF and eqUF beams and (bottom) on symmetry of WF beams for five years and eqUF beam for 1.5 years.

The long‐term (five‐year) stability of the eqUF beam was comparable to that of the WF beam. The top panel of Fig. 6 shows the differential histogram of the machine output offset for both the WF and the eqUF beams. The output failed the 3% tolerance over a five‐year period only five times for the WF beam (1386 data points), and three times for the eqUF beam (1354 data points). The overall average dose output offset was 0.60% for both beams. The standard deviation was 1.38% for the WF beam and 0.99% for the eqUF beam, an insignificant difference (p=0.3852). The differential histogram of symmetry offsets in both cross‐plane (CP) and in‐plane (IP) directions over a five‐year period for the WF beam and 1.5 years for the eqUF beam is seen in the bottom panel of Fig. 6. Overall, the WF beam showed a smaller percentage of data points out of the 3% tolerance: 4.3% in CP symmetry and 3.9% in IP symmetry. The corresponding results for the eqUF beam were 9.5% and 12.9% in CP and IP symmetry, respectively. The higher percentage of days exceeding tolerance for the eqUF beam is expected for symmetry, since this beam is far more sensitive to small variations in detector setup.

**Figure 7:**
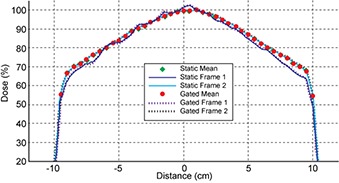
Profiles of eqUF beam with 0.5 cm spatial resolution during a delivery of 100 MU in the static or 500 ms gated mode.

**Figure 8:**
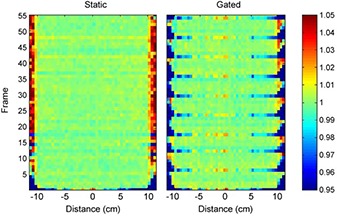
Percent differences in the profile of each frame in static or 500 ms gated mode compared to the average profile.

The eqUF beam dose profiles recorded by the linear diode array demonstrated beam stability within 0.144 s, as shown in Fig. 7. The figure shows the first and second frame (0.072 s per frame) compared to the average profile over the static or gated delivery (0.5 s gating window) of 100 MU. The profile in the first 0.072 s (Frame 1) differs from the average profile in both the static and gated delivery modes. But both profiles converge on the average profiles an additional 0.072 s after the beam turned on (Frame 2). Figure 7 shows that the average profiles under static delivery and 0.5 s gating mode are very similar. Figure 8 shows the percent difference between profiles of each frame and the overall average profile. In static mode, almost all points were within 2% after the first 0.072 s, except for the peripheral points that were in the beam penumbra. In the 0.5 s gating mode, a pattern of a profile similar to the first frame profile in static mode, followed by profiles with differences well within 2% occurred repeatedly in every 6 to 7 frames. This corresponds to the period of the simulated trace used to gate the beam.

## IV. DISCUSSION

The most attractive property of the UF beam is the increase in dose rate. If the UF beam is obtained simply by removing the flattening filter without any other change in beam control parameters, the dose rate of the energy unaltered UF beam was increased twofold. This is consistent with data reported by various other groups.^(^ ^9^ ^,^ ^10^ ^,^ ^12^ ^,^ ^24^ ^)^ But when the UF beam energy was adjusted so that the beam quality matches that of the original WF beam, the dose rate increased fivefold compared to the WF beam. This indicated that another two‐and‐a‐half‐fold increase in dose rate was a result of the increase in beam energy alone by quality matching. This is due to the design of the Siemens accelerating wave guide, which is made to be most efficient at a specific energy, which in our case is 15 MeV. The eqUF beam energy is higher than the UF beam and closer to 15 MeV. The accelerating wave guide is more efficient at the eqUF beam energy level so that the output is greater in the eqUF beam case.

While the eqUF beam shares various beam properties with the UF beam such as increased dose rate, the eqUF beam has other unique beam properties. In contrast with the observation that $d_{\max}$ of the energy unaltered UF beam shifted slightly towards the surface,^(^ ^10^ ^)^ $d_{\max}$ of the eqUF beam shifted slightly deeper. This is expected, since the effect of increased weight in low‐energy photons is shallower $d_{\max}$, as in the energy unaltered UF beam. In the eqUF beam, the increased photon energy results in more penetrating photons whose effect is deeper $d_{\max}$. When the latter effect exceeds the effect of increased weight in low‐energy photons, the overall effect is deeper $d_{\max}$, as in the eqUF beam. The penumbra of the energy unaltered UF beam decreased slightly due to reduced head scatter, as also observed by other groups. But the penumbra of the eqUF beam increased slightly. This may be due to the increased ability of partially traversing the collimator and resulting in a larger penumbra for photon beams with increased energy. When this effect exceeds the effect of reduced head scatter, the overall penumbra is larger, as observed in the eqUF beam.

The variation of collimator and phantom scatter associated with changes in field sizes decreased in the eqUF beam as a result of removal of the flattening filter, a major source of scatter radiation. The projection area on the flattening filter shaped by field size from the point's–eye view determines the amount of scatter.^(^ ^27^ ^)^ The large variation in WF beam dose profiles with depth is a result of the existence of the flattening filter, which can only optimize the flatness of the profile at a specific depth, usually a depth of 10 cm. An attribute of filter removal is less OAR variation, a characteristic shared by both the eqUF and the energy unaltered UF beams.^(^ ^12^ ^,^ ^28^ ^)^ From this point of view, the eqUF is more consistent in the direction of the CAX.

Figure 4 reveals that the field size of the UF beam profiles identified with the method independent of the WF profiles were very similar to those in the WF beam profiles, even though the shape of the eqUF profiles differed greatly from the WF profiles. This indicates that the modification of the method originally presented by Pönisch et al.^(^ ^17^ ^)^ is suitable. As such, field sizes and penumbra measurements for UF beam profiles can be determined without relying on the WF beam profile.

The eqUF beam output was quite stable even at a much higher dose rate, but the symmetry exhibited an increased uncertainty, partly due to the much higher gradient in the cross‐axis profile. This makes it more sensitive to setup uncertainty of the measurement device. The long‐term beam stability data were extracted from daily quality assurance measurements. Thus, the increase in dose rate as a result of removing the flattening filter does not appear to result in beam instability. This is due to the reduced variation in beam steering, as noted in other studies.^(^ ^9^ ^)^ The instabilities of the beam profiles during the ramp‐up time are similar between static and gated delivery with both profiles becoming stable after 0.072 s. It is feasible to use the eqUF beam in gated mode even with gating windows as small as 0.5 s.

## V. CONCLUSIONS

A much greater dose‐rate increase was observed in the eqUF beam on a dual energy standing‐wave linear accelerator with beam characteristics similar to those reported in the literature. A generalized method for defining field size based on the dose profiles of the UF beam without relying on WF beam data is presented. The eqUF beam dose output has proven stable over five years and has a dose profile that is stable after 0.072 s, which is comparable to the WF beam stability.
